# Sentido de coherencia en universitarios: recurso para el cuidado en tiempos de pandemia

**DOI:** 10.15649/cuidarte.2435

**Published:** 2023-03-31

**Authors:** Consuelo Vélez Álvarez, Natalia Sánchez Palacio, Diana Paola Betancurth Loaiza

**Affiliations:** 1 . Universidad de Caldas, Facultad de Ciencias para la Salud, Departamento de Salud Pública, Manizales - Colombia. Email: diana.betancurth@ucaldas.edu.co Universidad de Caldas Universidad de Caldas Colombia diana.betancurth@ucaldas.edu.co; 2 . Universidad de Caldas, Facultad de Ciencias para la Salud, Departamento de Salud Pública, Manizales - Colombia. Email: natalia.sanchez@ucaldas.edu.co Universidad de Caldas Universidad de Caldas Colombia natalia.sanchez@ucaldas.edu.co; 3 . Universidad de Caldas, Facultad de Ciencias para la Salud, Departamento de Salud Pública, Manizales - Colombia. Email: consuelo.velez@ucaldas.edu.co Universidad de Caldas Universidad de Caldas Colombia consuelo.velez@ucaldas.edu.co

**Keywords:** Cuidado, Estudiantes, Impacto Psicosocial, Epidemia, Coronavirus., Caregiving, Students, Psychosocial Impact, Epidemic, Coronavirus., Cuidado, Estudantes, Impacto Psicossocial, Epidemia, Coronavírus.

## Abstract

**Introducción:**

El constructo Sentido de Coherencia derivado del modelo salutogénico, permite afrontar factores estresantes a través de activos para mantener una buena salud.

**Objetivo:**

Describir el Sentido de Coherencia (SOC-29) en estudiantes universitarios de la ciudad de Manizales como recurso de cuidado en tiempos de pandemia.

**Materiales y Métodos:**

Se realizó un estudio cuantitativo, descriptivo con una fase asociativa y comparativa, con 566 estudiantes matriculados en universidades públicas y privadas de la ciudad de Manizales, Caldas (Colombia), durante el primer período académico del 2021. La técnica de recolección fue la encuesta online. Para el análisis univariado se realizó distribución de frecuencias, medidas de tendencia central y dispersión, y para el bivariado U de Mann Whitney, H de Kruskal-Wallis y Chi-cuadrado.

**Resultados:**

El valor del Sentido de Coherencia osciló entre 58 y 185 puntos, promedio 133.6 ± 24,4 puntos, un 27% de los participantes informó la pandemia afectó negativamente su vida, se encontró asociación estadísticamente significativa p<0,05 entre el cambio en las condiciones de vida y todos los ítems de la escala.

**Conclusiones:**

La consolidación del Sentido de Coherencia como parte integral de la salud mental de los universitarios los prepara para afrontar cambios en sus condiciones de vida y hace factible direccionar acciones de cuidado en el contexto social, familiar y académico.

## Abstract (en)

**Introduction:**

The construct of the sense of coherence, derived from the salutogenic model, allows people to cope with stressors through resources to maintain good health.

**Objective:**

To describe the sense of coherence (SOC-29) among university students in the city of Manizales as a care resource in times of pandemic.

**Materials and Methods:**

A quantitative and descriptive study with an associative-comparative phase was conducted with 566 students enrolled in public and private universities in Manizales, Caldas, (Colombia), during the first semester of 2021. The data collection technique was online surveys. For the univariate analysis, frequency distribution and central tendency and dispersion measures were determined. For bivariate analysis, the Mann-Whitney U test, Kruskal-Wallis H test, and Chi-square test were used.

**Results:**

The SOC-29 score ranged from 58 to 185 points (mean 133.6 ± 24.4 points), and 27% of the participants reported that the pandemic negatively affected their life, a statistically significant association (p<0.05) was found between the change in living conditions and all the items of the scale.

**Conclusions:**

Consolidating the sense of coherence as an integral part of university students' mental health prepares them to cope with changes in their living conditions and makes it feasible to direct care actions in social, family, and academic settings.

## Resumo

**Introdujo:**

A construgáo Sentido de Coerencia derivada do modelo salutogénico, permite lidar com os estressores através de ativos para manter a boa saúde.

**Objetivo:**

Descrever o Sentido de Coerencia (SOC-29) em estudantes universitários da cidade de Manizales, Caldas (Colombia), como um recurso para o cuidado em tempos de pandemia.

**Materiais e Métodos:**

Foi realizado um estudo quantitativo, descritivo com uma fase associativa e comparativa com 566 estudantes matriculados em universidades públicas e privadas na cidade de Manizales, Caldas (Colombia), durante o primeiro período academico de 2021. A técnica de coleta foi a pesquisa on-line. Para a análise univariada, foram utilizadas a distribuido de frequéncia, medidas de tendencia central e dispersáo, e para a análise bivariada foram utilizadas Mann Whitney U, Kruskal-Wallis H e Chi-quadrado.

**Resultados:**

O valor de senso de coeréncia variou de 58 a 185 pontos, média 133,6 ± 24,4 pontos, 27% dos participantes relataram que a pandemia afetou negativamente suas vidas, associagáo estatisticamente significativa p<0,05 foi encontrada entre mudanza nas condigóes de vida e todos os itens da escala.

**Condusdes:**

A consolidado do Senso de Coeréncia como parte integrante da saúde mental dos estudantes universitários os prepara para enfrentar mudangas em suas condigóes de vida e torna viável a abordagem de agóes de cuidado no contexto social, familiar e académico

## Introducción

Al analizar los factores que aportan a mantener el bienestar psicológico y la salud física surge el constructo de Sentido de Coherencia (SOC) propuesto por Antonovsky, que aborda la interacción entre ambos conceptos desde la teoría salutogénica^1^, este se asume como un recurso personal para afrontar situaciones difíciles, por lo que está considerado como una fuente que reduce el estrés y promociona la vida saludable.

El sentido de coherencia conduce a evaluar las circunstancias de la vida en tres dimensiones como comprensibles, manejables y significables. La primera la comprensibilidad, relacionada con la manera en que los eventos de la vida son predecibles, coherentes y estructurados; la manejabilidad, hace relación a la forma en que las personas disponen de capacidades y recursos para afrontar la cotidianidad, y la tercera dimensión es la motivación para convertir lo realizado en satisfactorio y con significado para la vida, además, a la orientación positiva de la vida denominada significatividad^1^^,^^2^.

En contraste, la actual pandemia que se vive en el mundo ocasionada por el síndrome respiratorio agudo severo SARS-CoV-2 (COVID 19) que se originó en la ciudad de Wuhan, China a fines de diciembre de 2019 y se ha convertido en la amenaza para la salud pública más grave del siglo actual con 190.355.867 casos confirmados y 4.088.069 muertes reportadas (cifras a 18 de julio de 2021^3)-(^^5^.

Esta emergencia sanitaria ha sido un hecho social que ha afectado todas las dimensiones de la vida humana, incluso la familiar^6^, como causa del enfrentamiento directo de las personas a situaciones entre ellas, el temor a la muerte, situación económica compleja, el sedentarismo, el estrés, la soledad, la infoxicación, adaptaciones a los modelos educativos, laborales y alteraciones de salud mental sobre todo en los jóvenes.

Para Eva Millet^7^ el confinamiento ha determinado que la mayoría de los jóvenes se enfrenten a nuevas demandas, adversidades y por ende transformaciones o adaptaciones inesperadas derivadas de pasar las 24 horas diarias con sus padres, la capacidad de agencia en la socialización, respecto a las Tablet, a las TIC y las nuevas dinámicas con los grupos de pares como consecuencia de un patrón inesperado, prolongado y repentino que ha modificado sus modos de vida.

Por ende, el rol del sentido de coherencia en tiempos de pandemia cobra relevancia configurándose como herramienta/recurso individual para los jóvenes en la transformación positiva de los estilos de vida. En la perspectiva del cuidado, puede decirse que desde esta se aborda de manera integral aspectos relacionados con las conductas, motivaciones y sentido de confianza interiorizado para aprovechar sus recursos internos y del entorno inmediato^8^. Así, las personas con alto puntaje de SOC hacen uso de sus Recursos Generales de Resistencia -RGS- (recursos materiales, biológicos/mentales, emocionales, físicos, existenciales, entre otros), los movilizan para resolver dificultades y para superar el estado emocional, en este caso los jóvenes presentarían una mayor habilidad (SOC fuerte) para evitar o prevenir situaciones riesgosas^9^^,^^10^.

Al respecto de este abordaje salutogénico con estudiantes universitarios, se han documentado estudios para evidenciar que el sentido de coherencia y algún acontecimiento crítico están relacionados, por ejemplo, el cansancio emocional por periodo de estudio^11^, consumo de alcohol^12^^,^^13^ y el apego,^14^ y de esta manera el sentido de coherencia podría ser una orientación general para que las personas aumenten la probabilidad de que estas situaciones se resuelvan. Estudios como el de Escobar y cols^15^ y Guerrero^16^, relacionan el estilo de vida promotor de salud y el sentido de coherencia.

En cuanto a la vida cotidiana de jóvenes universitarios en tiempo de pandemia, existen estudios en México que exploraron la salud mental y las emociones^17^, en España abordaron los efectos sobre las relaciones familiares^18^ y en España, Italia y Ecuador han estudiado el impacto de la virtualidad^19^, en los cuales se identificaron condiciones de miedo, incertidumbre, tristeza, estrés, fatiga y en general cambios en el estado anímico a raíz de los sucesos acontecidos por el coronavirus, entre los que se encuentran la educación virtual, las altas tasas de morbimortalidad, el desempleo, y las relacionades personales y familiares.

Ante este panorama, es necesario explorar la relación del sentido de coherencia en poblaciones que están experimentando diferentes emociones relacionadas con el contexto de la pandemia. Por ello, el objetivo de este estudio es describir el Sentido de Coherencia (SOC-29) en estudiantes universitarios de la ciudad de Manizales como recurso de cuidado en tiempos de pandemia.

## Materiales y Métodos

Se realizó un estudio de diseño cuantitativo, descriptivo con una fase asociativa, a través del cual, se indagó por el sentido de coherencia y la manera como este se vio afectado en el marco de la pandemia por SARS-CoV-2 (COVID-19).

La población estuvo constituida por el total estudiantes matriculados en 2 universidades públicas y 3 universidades privadas de la ciudad de Manizales (40.000 estudiantes), Caldas (Colombia) de los cuales participaron en total 566 estudiantes.

Se consideraron como criterios de inclusión: ser estudiante de pregrado, de cualquier programa; contar con matrícula activa en alguna de las universidades del municipio de Manizales y aceptar de manera voluntaria participar en el estudio.

La recolección de la información se llevó a cabo durante el primer período académico de 2021, a través de un formulario de Google que contenía el cuestionario SOC de 29 ítems, además de información sociodemográfica, que fue remitido por jefaturas de investigación de cada universidad participante.

La Escala SOC-29 creada por el autor Aaron Antonovsky evalúa la capacidad que tienen las personas para aprovechar los recursos disponibles en sus entornos inmediatos y distantes, y considerarlos como insumos valiosos para enfrentar los estímulos presentes a lo largo de la vida^20^. El instrumento validado para población colombiana (Alfa de Cronbach 0,838 e Índice de Validez de Contenido 0,83) consta de 29 preguntas tipo Likert valoradas en una escala de 1 a 7, donde entre más alto sea el valor de la sumatoria final de todos los ítems, más alto es el nivel de sentido de coherencia. A través de esta se miden tres dominios cognitivos: la comprensibilidad, capacidad de las personas de comprender como está organizada su vida (11 preguntas), la manejabilidad, capacidad de las personas para manejar sus vidas (10 preguntas) y la significatividad, capacidad de las personas de sentir que la vida propia está orientada hacia metas que desean alcanzar (8 preguntas^20)^.

Al ingresar los estudiantes encontraban la información sobre el proyecto, su objetivo y el respectivo consentimiento informado, si aceptaban participar en el estudio de manera voluntaria avanzaban en el diligenciamiento del mismo. El tiempo estimado para completar el instrumento fue de 10 minutos.

El proceso de envío por las Universidades se repitió en los meses de febrero, marzo y abril, tiempo en el cual las investigadoras realizaron corte y limpieza de bases de datos y de esta manera en el mes de mayo se dio inicio al análisis de la información.

Los datos de la investigación fueron depurados y organizados en el programa Excel versión 2010, mismos que fueron validadores y puestos a disposición pública en el Data-set. A partir de esta base la información se tabuló y analizó en el programa SPSS versión 22.0 licenciada por la Universidad de Caldas^21^. Para el análisis de información descriptiva se emplearon medidas de distribución de frecuencias, de tendencia central y de dispersión según la naturaleza de las variables. Adicionalmente, en la fase asociativa (con las variables de la escala y la edad, sexo, trabajo, tipo de universidad, estado civil, estrato socioeconómico, régimen de afiliación y cambios condiciones de vida) teniendo en cuenta el comportamiento y el tipo de variables se aplicaron las pruebas de Chi cuadrado, U de Mann Whitney y H de Kruskal-Wallis. En todos los casos se consideró como nivel de significancia estadística el valor de probabilidad p< 0,05.

El proyecto fue avalado por el Comité de Bioética de la Universidad de Caldas con el consecutivo cbcs-003. Según la Declaración de Helsinki de la Asociación Médica Mundial el trabajo realizado cumplió con los parámetros enunciados en ella, encaminados a la protección de los participantes^22^. En el marco de la Resolución 008430 del Ministerio de Salud Colombiano, en su artículo 11, se consideró como “investigación sin riesgo”^23^. Cada estudiante que aceptó participar en el estudio diligenció el consentimiento informado, los participantes tenían la autonomía para retirarse en cualquier parte del proceso o finalizar el cuestionario.

## Resultados

Participaron 566 estudiantes de universidades públicas y privadas (Tabla 1), la edad osciló entre 17 y 35 años, con una media de 22,19 años ± 2,36 años. El 66,10% fueron mujeres y el 95,60% manifestaron estar solteros en el momento del estudio. El estrato socioeconómico más común fue el tres, informado por el 36,90% de los pa rtici pantes, seguido del dos con un 25,40%. El 72,30% informó no trabajar actualmente. 511 estudiantes se encontraban cubiertos por el sistema de salud colombiano, de estos el 50.50% afiliados al régimen contributivo y el 39.80% al subsidiado. En lo relacionado a los convivientes, más del 70% expresó vivir con familiares, el 10% manifestó compartir casa con amigos o pareja y el 20% restante vivían solos o en residencias estudiantiles particulares e institucionales.

**Tabla 1:** Caracterización sociodemográfica

Característica	%(n) 566
Sexo	
Femenino	66.10 (374)
Masculino	33.90 (192)
Tipo de Universidad	
Pública	34,80 (197)
Privada	65,20 (369)
Estado civil	
Casado	1,40 (8)
Divorciado	0,40 (2)
Soltero	93,60 (530)
Unión libre	4,60 (26)
Característica	%(n) 566
Estrato socioeconómico	
0	1,10 (6)
1	15,90 (90)
2	25,40 (143)
3	36,90 (209)
4	13,40 (76)
5	3,90 (22)
6	3,40 (20)
Régimen de afiliación	50,50 (286)
Contributivo	49.50 (280)
Subsidiado	72,30 (409)
Trabaja	27,70 (157)
No	68,70 (389)
Si	8,30 (47)
Convivientes	
Familia	
Solo	
Pareja	3,90 (22)
Amigos	5,10 (29)
Residencias estudiantiles	14 (79)
Cambios en las condiciones de vida	27,00 (153)
Afectado negativamente	35,30 (200)
Mejorado	37,60 (213)
Permanecido igual	

Respecto al Sentido de Coherencia (SOC), el valor mínimo fue de 58 puntos y el máximo 185 con un promedio de 133,60 puntos ± 24,40 puntos. Los resultados del análisis del nivel de SOC, derivó en: nivel alto 392 estudiantes (69,30%); nivel moderado 76 (13,40%); nivel bajo 98 estudiantes (17,30%). Al indagar por los cambios en las condiciones de vida a raíz de la pandemia se encontró que para el 37,60% de los participantes permanecieron igual, el 35,30% expresó una mejora y un 27% informó que dicha contingencia generó afectaciones negativas en su vida.

La Tabla 2 da cuenta del nivel de sentido de coherencia por ítem y por dimensión valorado en los estudiantes. Nótese como todas las puntuaciones presentaron un valor mínimo de 1 y máximo 7 acorde a la escala definida en el instrumento. Para la dimensión de comprensibilidad en su orden se destaca los ítems 8, 5 y 6 como los de mejor valoración, de los once que la componen, con una media de calificación de 5,52 ±1.50, 4,93 ±1,56 y 4,88 ±1,55 respectivamente, mientras que los ítems 1, 2, 7 y 11 presentaron las calificaciones más bajas, con una media por debajo de 3.

Para la dimensión de manejabilidad evaluada a través de diez ítems de la escala, se evidenció que los ítems 18 y 15 presentaron las puntuaciones más altas, con una media 4,75 ±1,83 y 4,56 ±1,38 para cada uno, en contraste con los ítems 13, 14, 16 y 20 que tuvieron las valoraciones más bajas. Por último, en la dimensión de significatividad los ítems 22 y 28 presentaron las mejores calificaciones, elprimero con una media de 5,98 (±1,35), y para el segundo una media de 4,88 (±1,84), entre tanto, los ítems 23 y 27 obtuvieron las valoraciones más bajas de la dimensión (media inferior a 3).

**Tabla 2:** Sentido de Coherencia en universitarios.

Dimensión	Ítem	Media ± DE
Comprensibilidad	1. Cuando habla con las personas, ¿tiene la sensación de que no le comprenden lo que dice?	2,82±1,62
	2. Piense en las personas con las que tiene contacto diario, pero sin considerar a las más cercanas. ¿Qué tanto conoce a esas personas?	2,75±1,64
	3. ¿Alguna vez en el pasado le ha sorprendido el comportamiento de alguien a quien pensaba conocer muy bien?	4,37±1,44
	4. En los últimos diez años, su vida ha sido: llena de cambios sin que sepa qué pasará después/totalmente consistente y clara	3,41±1,73
	5. ¿Qué tan seguido se encuentra en una situación en la que no sabe qué hacer?	4,93±1,56
	6. Cuando enfrenta un problema, encontrar una solución es: siempre confuso y difícil/siempre claro y fácil	4,88±1,55
	7. Su vida en el futuro probablemente será: llena de cambios, sin saber qué pasará después/totalmente consistente y clara	2,84±1,78
	8. ¿Tiene ideas y sentimientos confusos?	5,53±1,51
	9. ¿Le ha sucedido que tiene alguna emoción que preferiría no sentir?	4,75±1,67
	10. ¿Con qué frecuencia siente que no tiene certeza de qué es lo que va a pasar en su vida?	3,85±1,88
	11. Cuando algo pasa, por lo general, se ha dado cuenta de que: exageró o minimizó su importancia/tomó las cosas en su debida proporción	2,07±1,23
Manejabilidad	12. En el pasado, cuando usted tuvo que hacer algo que dependía de la cooperación con otros, pensó que: lo lograría/ no lo lograría	4,29±1,58
	13. ¿Alguna vez se ha decepcionado de la gente en que confiaba?	2,07±1,21
	14. ¿Qué tan seguido piensa que recibe un trato injusto por parte de los demás?	2,72±1,76
	15. ¿Cómo describiría la forma en que usted ve la vida?	4,56±1,38
	16. Cuando algo desagradable sucedió en el pasado, su respuesta fue: preocuparse al máximo por ello/decir: “bueno, ya pasó, tengo que vivir con ello y seguir adelante”	2,86±1,33
	17. Cuando usted realiza algo que lo hace sentir bien, piensa que más tarde: seguro seguirá sintiéndose bien/seguro sucederá algo que eche a perder lo bien que se siente	4,42±1,79
Dimensión	Ítem	Media ± DE
	18. ¿Cree que en el futuro siempre habrá personas con las que podrá contar?	4,75±1,83
	19. Mucha gente a veces siente que fracasa en ciertas situaciones. ¿Con cuánta frecuencia se ha sentido así en el pasado?	3,99±1,77
	20. Cuando piensa en las dificultades que probablemente tendrá que enfrentar en aspectos importantes de su vida, tiene la impresión de que: siempre tendrá éxito para superar las dificultades/no tendrá éxito para superar las dificultades	2,80±1,73
	21. ¿Con qué frecuencia siente que no podrá mantener sus emociones bajo control?	3,53±1,78
Significatividad	22. ¿Con qué frecuencia piensa que no le importan las cosas que pasan a su alrededor?	5,98±1,35
	23. Para usted la vida: está llena de interés/es completamente rutinaria	2,52±1,70
	24. Hasta ahora su vida ha tenido: metas poco claras/metas y propósitos muy claros	4,21±1,78
	25. La mayoría de las cosas que usted hará en el futuro probablemente serán: totalmente fascinantes/totalmente aburridas	4,19±1,51
	26. Cuando piensa sobre su vida, con frecuencia: siente lo bueno que es estar vivo/se pregunta para qué vive	4,14±1,71
	27. Hacer lo que hace a diario es: siempre fuente de placer y satisfacción/siempre una fuente de dolor y aburrimiento	2,69±1,37
	28. Usted presiente que su vida personal en el futuro: no tendrá ningún significado ni propósito/estará llena de significado y propósito	4,88±1,84
	29. ¿Con qué frecuencia tiene la impresión de que las cosas que hace en su vida diaria son insignificantes?	4,40±1,87

En el análisis bivariado al comparar la calificación por ítem de la escala SOC y las características sociodemográficas de los participantes, cuyos resultados se presentan en la Tabla 3, se encontró que el tipo de universidad, el sexo y la edad fueron las características sociodemográficas que evidenciaron asociaciones estadísticamente significativas (p<0,05) con una mayor cantidad de ítems de la escala (17/29 ítems, 11/29 ítems y 8/29 ítems respectivamente).

**Tabla 3:** Sentido de Coherencia (SOC-29) y Características sociodemográficas

Ítem escala SOC	Edad	Sexo	Trabajo	Tipo de universidad	Estado civil	Estrato socioeconómico	Régimen de afiliación	Cambios condiciones de vida
	p*	p*	p*	p*	p*	p**	p*	p**
1	.94	.35	.86	.00	.57	.60	.24	.00
2	.82	.28	.70	.28	.56	.32	.53	.01
3	.42	.30	.80	.00	.43	.84	.43	.01
Ítem escala SOC	Edad	Sexo	Trabajo	Tipo de universidad	Estado civil	Estrato socioeconómico	Régimen de afiliación	Cambios condiciones de vida
	p*	p*	p*	p*	p*	p**	p*	p**
4	.11	.04	.27	.50	.20	.22	.62	.01
5	.76	.02	.40	.00	.93	.96	.16	.01
6	.93	.01	.07	.39	.21	.99	.23	.00
7	.36	.45	.00	.19	.98	.03	.68	.00
8	.77	.31	.13	.00	.69	.12	.75	.00
9	.50	.06	.29	.54	.83	.11	.61	.00
10	.54	.18	.86	.07	.86	.50	.05	.00
11	.58	.83	.51	.01	.32	.08	.53	.00
12	.04	.02	.16	.54	.03	.20	.70	.00
13	.49	.03	.06	.28	.29	.01	.25	.00
14	.02	.51	.11	.00	.35	.42	.37	.00
15	.29	.00	.07	.01	.19	.38	.80	.00
16	.05	.62	.41	.00	.50	.05	.75	.00
17	.04	.75	.34	.55	.56	.24	.41	.00
18	.08	.00	.10	.01	.06	.82	.88	.00
19	.00	.57	.00	.04	.02	.74	.50	.00
20	.24	.62	.17	.30	.67	.09	.22	.00
21	.12	.14	.06	.06	.62	.52	.23	.00
22	.30	.91	.08	.00	.77	.08	.46	.00
23	.74	.54	.42	.00	.62	.28	.78	.00
24	.00	.80	.15	.00	.15	.65	.21	.00
25	.77	.01	.05	.03	.94	.72	.50	.00
26	.01	.00	.24	.74	.19	.42	.79	.00
27	.03	.28	.15	.01	.13	.44	.64	.00
28	.20	.33	.98	.01	.68	.26	.44	.00
29	.50	.00	.42	.19	.23	.61	.36	.00

Adicionalmente, se comparó la calificación de cada ítem de la escala SOC con la respuesta a los cambios de las condiciones de vida a raíz de la pandemia por COVID-19, encontrándose una asociación estadísticamente significativa (p<0,05) en los 29 ítems que evalúa la escala, lo que refleja la relación entre estas dos variables (Tabla 3). De igual modo, en esta misma tabla, se evidenciaron diferencias estadísticamente significativas (p<0,05) entre todos los ítems de las tres las dimensiones de la escala (comprensibilidad, manejabilidad y significatividad), y los cambios de vida en tiempos de pandemia.

En la Tabla 4 se presentan los resultados de la asociación de las características sociodemográficas con las dimensiones del SOC y el nivel de SOC total. Al respecto se puede apreciar para la dimensión de Comprensibilidad que las puntuaciones medias de todas las características oscilaron entre 39 y 53, con un mínimo de 11 y un máximo de 77 según lo definido en el instrumento, variación semejante a la de Manejabilidad, quien cuenta con un mínimo de 10 y un máximo de 70 puntos. Por su parte, la dimensión de Comprensibilidad presentó valores de media superiores, si se considera que su escala de calificación total puede variar entre 8 y 56, y para este caso las medias de la mayoría de las variables se ubicaron por encima de 40. Finalmente, en relación con el SOC total cuyo puntaje máximo es 203, se encontraron marcadas variaciones en todas las mediciones sociodemográficas, oscilando entre 115,59±21,53 y 150,12±18,97.

**Tabla 4:** Características sociodemográficas y dimensiones del SOC*

Característica	Comprensibilidad	Manejabilidad	Significatividad	SOC total
Sexo				
Femenino	44,85±10,87	45,05±6,57	42,33±8,94	131,60±24,65
Masculino	47,08±10,46	47,47±6,83	42,78±8,52	137,43±23,50
Edad				
Menores 24 años	45,05±10,65	45,68±6,59	42,20±8,90	132,51±24,25
25 años o más	47,92±11,01	46,65±7,34	43,69±8,29	138,03±24,67
Tipo de Universidad				
Pública	44,07±10,98	45,06±7,03	40,48±10,16	128,84±26,86
Privada	46,43±10,58	46,30±6,56	43,55±7,77	136,11±22,62
Estado civil				
Casado	52,25±7,80	49,38±6,59	48,25±6,45	150,12±18,97
Divorciado	43,50±17,68	46,50±2,12	48,50±3,54	137,50±27,58
Soltero	45,48±10,76	45,75±6,72	42,43±8,77	133,27±24,42
Unión libre	46,27±11,34	47,11±7,56	41,50±9,63	134,42±24,93
Régimen de afiliación				
Contributivo	46,90±10,24	46,49±6,30	43,24±8,43	136,56±22,96
Subsidiado	44,30±11,19	45,37±7,25	41,67±9,38	130,69±25,77
Trabaja				
No	45,03±11,09	45,57±6,83	42,03±9,09	132,11±25,04
Si	47,11±9,78	46,65±6,49	43,68±7,87	137,41±22,28
Característica	Comprensibilidad	Manejabilidad	Significatividad	SOC total
Convivientes				
Familia	45,22±10,62	45,57±6,73	42,09±8,74	132,29±24,25
Solo	48,79±11,27	46,45±6,36	44,80±9,13	140,68±24,83
Pareja	47,67±10,54	42,15±6,86	47,87±8.18	135,87±25,25
Amigos	46,48±10,24	47,10±6,46	44,45±6,61	138,17±20,97
Residencias estudiantiles	44,15±11,06	45,98±7,26	41,43±9,70	131,16±25,79
Cambios en el estilo de				
vida				
Afectado negativamente	39,06±8,82	41,49±6,32	36,78±8,93	115,59±21,53
Mejorado	48,55±9,88	48,26±5,60	46,58±6,04	143,48±19,11
Permanecido igual	47,55±10,91	46,77±6,58	42,74±8,69	137,20±23,94

Finalmente, se analizó la asociación entre las características sociodemográficas de los participantes y los cambios de vida durante la pandemia, encontrando solo una asociación estadísticamente significativa con la edad, con un valor de Chi-Cuadrado de 6,429 (p=0,040).

## Discusión

Desde la corriente sociocultural la edad es una condición necesaria pero no suficiente para tener juventud^24^. Bourdieu afirma que está etapa de la vida es una construcción social, histórica, cultural y relacional permeada por circunstancias culturales, sociales, económicas y políticas^25^. En este sentido, los jóvenes y en este caso los universitarios asumen un papel activo en la construcción y consolidación de este momento vital, por tanto, es necesario reconocer que ellos se encuentran sometidos a diversas tensiones y situaciones que generan riesgos en su contexto de vida^24^. Para fortalecer la capacidad de afrontamiento, el Sentido de Coherencia (SOC) adquiere gran importancia en la identificación de oportunidades y necesidades, al configurarse como factor que actúa de escudo en la iniciación y mantenimiento de conductas negativas para la vida y la salud, y en la posibilidad de reforzar acciones de autocuidado^26^.

El nivel de SOC en la mayoría de los estudiantes participantes de este estudio fue alto, lo que da cuenta de su capacidad de afrontamiento y resiliencia ante situaciones difíciles. Los resultados coinciden con los hallados por Escobar y colaboradores, quienes valoraron el estilo de vida promotor de salud y el sentido de coherencia en 300 jóvenes universitarios mexicanos, identificaron que más del 50% de estos presentaron un elevado nivel de SOC^15^. Adicional a ello, en este mismo artículo se encontró que la media de todos los ítems y dimensiones de la escala presentaron puntuaciones altas, aspectos consistentes con los encontrados en esta investigación^15^.

El estudio de la capacidad de afrontamiento en los estudiantes universitarios expresada a través del sentido de coherencia y la manera como la situación actual a nivel mundial afecta a esta población, se debe convertir en herramienta de trabajo para que desde la perspectiva del cuidado y en el marco de un trabajo interdisciplinario las universidades diseñen y fortalezcan estrategias para consolidar esta capacidad en dicha población, dado que estudios como el de Cobo y Zapata et al llaman la atención sobre el desarrollo de acciones en salud mental para prevenir posibles efectos psicológicos de la COVID-19^27^^,^^28^.

En el marco de la pandemia por SARS-CoV-2 (COVID-19), diferentes estudios han abordado características emocionales y de salud mental en población universitaria algunos resultados difieren de los encontrados en la presente investigación, ya que reportan altos niveles de tristeza, fatiga, stress y ansiedad con una posible baja capacidad de afrontamiento ante situaciones de crisis^17^^,^^29^^,^^30^, en contraste, el estudio de Tardivo realizado también en el marco de la pandemia, reporta resultados similares a los del presente estudio, informó que la mitad de los estudiantes no tuvieron cambios, otros los reportan como positivos y en menor porcentaje negativos^18^.

Se resalta la asociación estadísticamente significativa encontrada entre el SOC y la variable tipo de universidad. Esta asociación puede ser explicada por el solo periodo transicional en el que se encuentran los jóvenes universitarios por lo que es importante favorecer actitudes y prácticas positivas para mantener la salud (consumo de frutas y verduras, ejercicio, uso del tiempo libre, las viviendas), no obstante, esta situación está mediada por los recursos económicos y las oportunidades para fortalecer sus activos^31^.

Autores como Sainz^32^ y Malagón-Aguilera y cols^33^ encontraron la presencia de dificultades económicas en jóvenes como un factor de exposición que aumenta la probabilidad de causar daño e influir negativamente en las condiciones de vida; En contraste, unos recursos socioeconómicos satisfactorios desde la perspectiva salutogénica son factores protectores, lo cual favorece el desarrollo y la resistencia ante situaciones adversas. De otro lado, el trabajo desarrollado por Broche y cols, pone en evidencia como las dificultades económicas pueden ser la principal causa de alteraciones psicológicas frente a situaciones que generan estrés^34^. Esto, soporta lo hallado en la presente investigación, donde las condiciones de vida de los participantes se vieron altamente afectadas a raíz de la pandemia, principalmente en lo relacionado con las condiciones socioeconómicas.

En lo relacionado a los cambios en las condiciones de vida durante la pandemia, los hallazgos del análisis bivariado mostraron asociaciones estadísticamente significativas con todos los ítems de la escala. Para analizar esta relación es necesario retomar el resultado inicial del SOC en el que la mayoría de los participantes quedó clasificada en nivel alto. En este sentido, tal como lo reporta Guevara, cuando se aplica la escala y se valora como alta, podría reflejar una buena salud y considerarse protector para patologías mentales como depresión, ansiedad, estrés entre otras, otro aspecto relevante en el marco del cuidado es que quienes tienen altos puntajes son personas de mayor auto control, optimismo y capacidad para afrontar los problemas^35^.

Al respecto, estudios desarrollados en estudiantes universitarios han evidenciado como el cuidado y la salud mental interactúan y se expresan en mejores condiciones de vida, por tanto, debe ser un compromiso de las instituciones de educación superior trabajar sobre esta dimensión vital^36^. Ribot, Chan y González^37^ resaltan la importancia de potenciar los niveles de resiliencia y el cuidado en las poblaciones vulnerables entre ellas los jóvenes, dado que su consolidación minimiza el impacto psicosocial en situaciones difíciles.

Las autoras reconocen como principal limitación del estudio la situación vivida en el momento del mismo que impidió contar con un mayor número de participantes. Los resultados de este trabajo pueden ser aprovechados por los programas dirigidos al bienestar de los estudiantes de acompañamiento psicosocial que generen intervenciones para transformar las condiciones de vida de los universitarios. Además, se convierten en insumo para el desarrollo de nuevas investigaciones en los que se puedan validar o aplicar programas que fortalezcan los niveles de resiliencia y la salud mental en la población universitaria.

## Conclusiones

Al analizar las diferentes dimensiones del instrumento se encontró que en la dimensión de comprensibilidad los ítems cinco, seis y ocho fueron los de mejor valoración, mientras que los ítems uno, dos, siete y once presentaron las calificaciones más bajas, con una media inferior a 3. En la dimensión de manejabilidad los ítems quince y dieciocho presentaron las puntuaciones más altas, en contraste con los ítems trece, catorce, dieciséis y veinte con bajas puntuaciones. En la dimensión de significatividad los ítems veintidós y veintiocho presentaron las mejores calificaciones, entre tanto, los ítems veintitrés y veintisiete obtuvieron las valoraciones más bajas de la dimensión (media inferior a 3).

El sentido de coherencia de los universitarios participantes en el estudio en su gran mayoría fue alto, esto hace factible la estructuración y desarrollo de estrategias y acciones de cuidado, las cuales deben ser coherentes con el contexto social, familiar y académico en el cual se materializa el proceso de aprendizaje.

Los hallazgos se convierten en insumos para que las instituciones de educación superior reconozcan la importancia de invertir en la salud mental de los estudiantes, esto permite fortalecer la capacidad de afrontamiento frente a situaciones de estrés. Por tanto, estrategias como consejerías, conformación de redes sociales de apoyo, y grupos de ayuda con los que el estudiante puede interactuar de manera constante, son alternativas para articular la diada salud mental y cuidado como eje fundamental para mejorar condiciones y calidad de vida.
